# Exploring perceptions of the educational environment among undergraduate physiotherapy students

**DOI:** 10.5116/ijme.53a5.7457

**Published:** 2014-07-19

**Authors:** Per J. Palmgren, Ingrid Lindquist, Tobias Sundberg, Gunnar H. Nilsson, Klara B. Laksov

**Affiliations:** 1 Department of Learning, Informatics, Management and Ethics, Karolinska Institutet, Sweden; 2 Department of Neurobiology, Care Sciences and Society, Karolinska Institutet, Sweden

**Keywords:** Physiotherapy, educational climate, educational environment, Dundee ready education environment measure, undergraduate

## Abstract

**Objectives:**

The aim of this study was to explore areas of strength and weakness in the educational environment as perceived by undergraduate physiotherapy students and to investigate these areas in relation to the respondents’ demographic characteristics.

**Methods:**

This study utilized a cross-sectional study design and employed the Dundee Ready Education Environment Measure, a 50-item, self-administered inventory relating to a variety of topics directly pertinent to educational environments. Convenience sampling was used, and the scores were compared across demographic variables. All undergraduate physiotherapy students in their first five terms of the programme in a major Swedish university were invited to participate in the study.

**Results:**

A total of 222 students (80%) completed the inventory. With an overall score of 150/200 (75%), the students rated the educational environment in this institution as “more positive than negative”. Two items consistently received deprived scores - authoritarian teachers and teaching with an overemphasis on factual learning. Students in term 4 differed significantly from others, and students with earlier university education experience perceived the atmosphere more negatively than their counterparts. There were no significant differences with regards to other demographic variables.

**Conclusions:**

This study provides valuable insight into how undergraduate physiotherapy students perceive their educational environment. In general, students perceived that their educational programme fostered a sound educational environment. However, some areas require remedial measures in order to enhance the educational experience.

## Introduction

The educational environment of professional health training is primarily shaped by the interactions between different stakeholder groups and the organizational structures of the environment. This environment occasionally referred to as “climate” or “atmosphere”, is complex, multifaceted, and can be described as the spirit and personality of an educational institution.^1^ Ideally, the educational environment should foster intellectual activities and academic progression while simultaneously encouraging friendliness, cooperation and support. Evaluating such an environment can be a complex endeavour because it may encompass a multitude of settings, features and stakeholders. Students comprise one of the key stakeholder groups, and research has shown that the educational environment heavily influences their behaviours and contributes to their learning, performance, contentment and success.^2^^-^^5^While the existing literature describes the importance of the educational environment, relatively little research has been conducted to explore the concept of what constitutes such environments. Although the word “environment” is synonymous with physical space (e.g. “surroundings” and “settings”), it also has social, emotional and intellectual connotations. The educational environment is primarily a theoretical construct that cannot be measured directly; however, it is manifested in students’ mundane experiences and perceptions, which can be assessed.To the best of our knowledge, only two studies have thus far endeavoured to identify and survey students perceptions of the educational environment in physiotherapy education. Brown et al.^6^ performed a cross-sectional study investigating different health science courses at an Australian university and found that undergraduate physiotherapy students generally held positive perceptions of their educational environment. Ousey et al.^7^ investigated the educational environment across six undergraduate healthcare courses in the United Kingdom, and their findings were comparable with those of Brown et al.^6^ However, both of these studies used very small samples and did not comprehensively investigate demographic variations. Consequently, there is a paucity of scientific research examining the educational environment of physiotherapy students compared to other health professions, such as medicine, dentistry, nursing and chiropractic, thereby resulting in a gap in the literature regarding how educational stakeholders in physiotherapy training perceive their educational environment.A variety of methodologies have been used to explore and quantify the presence of somewhat ethereal features of an educational environment, including qualitative,^8^ quantitative,^9^^-^^13^ and mixed-method^14^^,^^15^ paradigms. Many instruments are available to measure educational environments in undergraduate professional healthcare education, each of which has its own strengths and weaknesses in terms of design, validity and reliability. Arguably, the most widely used instrument is the Dundee Ready Educational Environment Measure (DREEM).^16^The DREEM inventory is an instrument that measures the perception of an educational environment and has been widely used in different educational contexts. It has been shown to have good psychometric properties with evidence based on test content (content validity) and internal structure (construct validity)^9^^,^^17^^,^^18^ and has consistently displayed good reliability in diverse settings.^4^^,^^19^^-^^25^ It has been used to explore, evaluate and compare the following dynamics: the educational environments of different institutions,^26^ students at various levels of training,^27^^-^^29^ institutions at different phases of curriculum reform^23^ and gender discrepancies.^30^^,^^31^ Thus, the measure has contributed to establishing a greater contextual understanding of professional healthcare education, including in relation to the broader healthcare community. In a recent systematic review,^16^ it was proposed that DREEM was likely to be the most suitable instrument for measuring the environment in undergraduate professional healthcare educational settings.In order to better understand the concept of the educational environment in undergraduate professional healthcare education, we previously investigated perceptions of the educational environment by applying the DREEM inventory to chiropractic students.^24^ The scarcity of similar studies which focus on physiotherapy students motivated us to investigate this group which, in many ways, is similar to chiropractic students, and investigate whether the findings may infer parallel trends for both vocational training settings.The aim of the current study was to explore areas of strength and weakness in the educational environment as perceived by undergraduate physiotherapy students and to investigate these in relation to the respondents’ demographic characteristics.

## Methods

### Study design

We implemented a cross-sectional study design. The study was part of a larger project which employed a prospective mixed-method multiple case study methodology; it was conducted within a pragmatic and interpretive research tradition. Ethical approval to conduct the study was obtained from the Regional Ethics Committee of Stockholm (2012/416-31/5). The completion of the DREEM inventory was undertaken on a voluntary basis, and none of the information collected was identiﬁable, thereby maintaining data anonymity. All the data was handled and stored in accordance with the tenets of the Declaration of Helsinki.

### Setting

The study was conducted at Karolinska Institutet in Stockholm, Sweden, a medical university offering a three-year, full-time undergraduate programme that culminates in a professional qualification and a Bachelor of Science degree in physiotherapy.

### Participants

A non-probability convenience sample of undergraduate physiotherapy students from five cohorts, terms 1-5, attending a traditional curriculum was invited to participate in the study. Students attending an individually adapted curriculum or other curricula were excluded. The DREEM inventory was administered during classes to ensure a high response rate. However, an electronic version was subsequently disseminated to improve the response rate. Participation was voluntary, and the questionnaire was anonymous.

### Instrument

The DREEM instrument has been translated and validated for use in Sweden.^23^ DREEM is a 50-item, self-administered, closed-ended inventory relating to a variety of topics directly pertinent to educational environments. Each item is scored by respondents from 4 to 0 with a 5-point Likert response as follows: 4= strongly agree; 3 = agree; 2 = unsure; 1= disagree and 0 = strongly disagree. Items with a mean score greater than 3.5 mainly represent strong areas; items with a mean score of less than or equal to 2 should be inspected more meticulously as they indicate problematic areas; and items with mean scores between 2 and 3 indicate areas that could be enhanced.^9^^,^^32^ The instrument has an overall score of 200, and we followed the interpretation guidelines provided by Lai et al.,^33^ and McAleer and Roff:^34^ 0 to 50 (0–25%) = very poor environment; 51 to 100 (26–50%)=plenty of problems in the environment; 101 to 150 (51–75%) = more positive than negative environment; and 151 to 200 (76–100%) = excellent environment.The items are allocated to five subscales based on students’ perceptions of the following: learning (SPL–12 items/maximum score 48); teaching (SPT–11 items/maximum score 44); academic self-perceptions (SASP – 8 items/maximum score 32); atmosphere (SPA – 12 items/maximum score 48) and social self-perceptions (SSSP – 7 items/maximum score 28).The items can be analysed on three levels: individually, pooled into five subscales and overall. Although the constructors of the DREEM instrument provide guidelines^34^ for its interpretation, they do not recommend appropriate methods for statistical inferences.The following open-ended question concludes the inventory: “Are there any other factors that you feel have an influence on the educational environment?”

### Data analysis

The completed surveys were manually entered into a Microsoft Excel data sheet and exported to the Statistical Package for the Social Sciences (SPSS) version 20.00 (IBM Corporation, Armonk, NY) for descriptive and inferential statistical analysis. As nine items (4, 8, 9, 17, 25, 35, 39, 48 and 50) from the instrument are negatively stated, corrections were made, thus resulting in higher scores designating disagreement with these items.Overall, subscale and individual scores were analysed if all items were completed by the respondents. Normal data distribution was assessed visually via boxplots by contrasting possible discrepancies among the parameters of central tendency, evaluating the skewness and kurtosis of the distributions and employing Kolmogorov-Smirnov and Shapiro-Wilk tests.The criterion variables were the perceptions of the educational environment as measured by the overall, subscale and individual scores of the DREEM inventory. The main predictor variables were term, gender, age, immigrant background (based on parents’ background), resitting exams, previous experience of higher education and intent upon completing the degree.Cronbach’s alpha was employed to assess internal consistency of the overall and subscale scores of the instrument, and a minimum coefficient alpha of 0.70 was used to indicate an adequate level of consistency.^35^ Non-parametric statistical tests were performed and selected to avoid influences of the distribution of the data. The Wilcoxon-Mann-Whitney test was used for ordinal data while the chi-square test was used to compare nominal data. The Kruskal-Wallis (one-way analysis of variance) test was used for independent between group analyses. P-values were adjusted for multiple comparisons by employing the Bonferroni correction of primary endpoints. Pearson’s correlation coefﬁcients were used to analyse correlations between the subscales. Probability values less than 0.05 were considered statistically signiﬁcant for all statistical tests.Responses to the open-ended question were transcribed verbatim, and the transcripts were examined line by line. Signiﬁcant sentences were identiﬁed, and central concepts were inductively grouped into emerging themes through a manifest content analysis^36^ by using an iterative process of going back and forth among original transcripts, signiﬁcant sentences and themes. We discussed the themes until we reached a consensus.

## Results

A total of 223 students completed the inventory, thereby yielding a response rate of 80%. One participant only responded to the demographic questions and was therefore excluded, thereby yielding a sample size of n = 222 for the data analysis. In term 1, 81% of the students completed the questionnaire. In terms 2 and 4, the response rates were 87% and 88%, respectively, and terms 3 and 5 recorded the lowest response rates with 72% and 73%, respectively. The respondents included 169 females (76%) and 53 males (24%), and the mean age was 24.7 (SD 5.8; ranging between 19 and 52). The demographics are presented in Table 1.

**Table 1:** Summary of demographic variables (n = 222)

Predictor variable	Level of variable	n (%)
Term	1 2 3 4 5	61 (28) 47 (21) 45 (20) 35 (16) 34 (15)
Gender	Female Male	169 (76) 53 (24)
Age	21 or younger 22–25 26 or older	60 (27) 102 (46) 60 (27)
Parents’ background	Both Swedish Swedish/other Both Nordic Nordic/other Both other	180 (81) 15 (7) 18 (8) 2 (1) 7 (3)
Accommodation	With parents Alone With a partner Other	49 (22) 61 (27) 82 (37) 30 (14)
Children	No Yes	205 (92) 17 (8)
Re-sitting exams*	Often From time to time Infrequently Never	1 (1) 19 (8) 82 (38) 113 (53)
Previous experience of higher education	No Yes	110 (50) 112 (50)
Intention upon completing degree^†^	Work Work with something else Study Work and study Other	142 (65) 2 (1) 14 (6) 55 (25) 7 (3)

*Seven missing values (n = 215)

^†^Two missing values (n = 220)

The overall reliability coefficient was high as alpha was 0.935 and did only increase marginally “if item deleted” for 3 of the 50 items. The subscales displayed alpha values at SPL 0.867, SPT 0.746, SASP 0.676, SPA 0.805, SSSP 0.633, and exceeded the threshold, except for SASP and SSSP. Overall, subscale and individual scoresThe overall score for the five cohorts was 150.0 (SD 21.9) out of 200 (75%), ranging from 73 “plenty of problems” to 200 “excellent.” SPA and SSSP generated the highest subscale scores (78%) while SASP produced the lowest subscale score (72%). The skewness estimate showed that the overall and subscale DREEM scores were negatively skewed. However, no values were less than -1, and the sample was fairly large. The overall and subscale scores are summarized in Table 2.

**Table 2:** DREEM subscale and overall scores for each of the terms presented as means and standard deviations (SD). Percentage of maximum score, minimum and maximum values, skewness of data and statistically significant differences are also displayed (n = 222).

Subscale /max score	Term 1 Mean (SD) n = 61	Term 2 Mean (SD) n = 47	Term 3 Mean (SD) n = 45	Term 4 Mean (SD) n = 35	Term 5 Mean (SD) n = 34	Total Mean (SD) n = 222	%max score	Min-max	Skewness	Significant difference between terms*
SPL/48	37.6 (5.1)	37.1 (6.3)	33.8 (7.5)	31.1 (7.4)	33.2 (5.0)	35.0 (6.7)	73	9-48	-0.94	1:4***; 1:5***; 2:4***
SPT/44	34.8 (3.7)	34.1 (5.1)	30.9 (5.9)	30.1 (4.6)	31.9 (5.1)	32.6 (5.2)	74	18-44	-0.60	1:3***; 1:4***; 2:4**
SASP/32	23.0 (3.6)	24.1 (3.4)	23.7 (4.3)	21.3 (5.1)	23.0 (3.8)	23.1 (4.1)	72	7-32	-0.49	NS*
SPA/48	38.5 (5.4)	38.3 (5.5)	38.5 (6.1)	34.7 (6.7)	36.4 (5.0)	37.6 (5.9)	78	20-48	-0.72	3:4**
SSSP/28	23.2 (3.1)	22.1 (3.4)	21.7 (3.6)	19.6 (4.1)	21.2 (3.7)	21.8 (3.7)	78	10-28	-0.61	1:4***
Overall/200	156.8 (17.7)	155.3 (22.0)	148.9 (23.8)	136.4 (23.7)	147.6 (17.3)	150.0 (21.9)	75	73-200	-0.79	1:4***; 2:4**
% max score	78	78	74	68	74	75				

*Non-Significant at 5%, **p < .01, ***p < .001. P-values adjusted for multiple comparisons using the Bonferroni correction

In the investigation of covariation among the subscales, the correlations between the SPL and the SPA were found to be high (Pearson’s correlation coefﬁcient 0.762, p=0.00). There was a fair degree of covariance for the other subscales (Pearson’s correlation coefﬁcient 0.553–0.717, p=0.00), thereby indicating dependent subscales.The total item mean amounted to 3.0 (SD 1.0). The highest mean score for an individual item was 3.6 (items 2 and 33), and six items scored 3.5 or above (items 2, 15, 19, 33, 40 and 46). The lowest observed mean score was 1.9 (items 9 and 25); however, these two were the only items scoring less than the expected mean. For these two items, less than 50% of the respondents agreed or strongly agreed, and more than 20% disagreed or strongly disagreed. Moreover, 32% of the items scored between less than 3.0 and the expected mean. Table 3 presents the scores for the individual items.

**Table 3:** Percentage-clustered categories, means and standard deviations (SD) for individual DREEM items (n = 222)

Items	n	Agree or Strongly agree %	Unsure %	Disagree or Strongly disagree %	Mean (SD)
1. I am encouraged to participate in class^1^	222	92	5	3	3.4 (0.7)
2. The teachers are knowledgeable^2^	221	96	2	2	3.6 (0.7)
3. There is a good support system for students who get stressed^5^	221	42	48	10	2.5 (1.0)
4. I am too tired to enjoy this course^5^*	222	60	11	29	2.5 (1.2)
5. Learning strategies which worked for me before continue to work for me now^3^	222	77	15	8	2.8 (0.9)
6. The teachers are patient with the patients^2^	209	67	32	1	3.0 (0.9)
7. The teaching is often stimulating^1^	222	88	8	4	3.1 (0.8)
8. The teachers ridicule the students^2^*	222	85	7	8	3.3 (1.0)
9. The teachers are authoritarian^2^*	221	37	20	43	1.9 (1.1)
10. I am confident about passing this year^3^	222	80	11	9	3.1 (1.0)
11. The atmosphere is relaxed during the clinical teaching^4^	219	81	11	8	3.0 (0.9)
12. This school is well timetabled^4^	222	72	16	12	2.8 (0.9)
13. The teaching is student-centered^1^	222	84	11	5	3.1 (0.8)
14. I am rarely bored in this course^5^	222	78	11	10	3.0 (1.0)
15. I have good friends in this school^5^	222	91	4	5	3.5 (0.8)
16. The teaching helps to develop my competence^1^	222	88	9	3	3.4 (0.8)
17. Cheating is a problem in this school^4^*	221	76	21	3	3.2 (0.9)
18. The teachers have good communication skills with patients^2^	208	67	31	2	2.9 (0.8)
19. My social life is good^5^	222	95	4	1	3.5 (0.6)
20. The teaching is well focused^1^	222	79	13	8	2.9 (0.8)
21. I feel I am being well prepared for my profession^3^	221	72	20	8	2.8 (0.9)
22. The teaching helps to develop my confidence^1^	222	79	15	6	3.0 (0.8)
23. The atmosphere is relaxed during lectures^4^	222	92	5	3	3.4 (0.7)
24. The teaching time is put to good use^1^	222	77	13	10	2.8 (0.9)
25. The teaching overemphasizes factual learning^1^*	221	32	29	39	1.9 (1.0)
26. Last year’s work has been good preparation for this year’s work^3^	214	68	26	6	2.9 (0.9)
27. I am able to memorize all I need^3^	221	53	28	19	2.4 (1.0)
28. I seldom feel lonely^5^	222	82	9	9	3.3 (1.0)
29. The teachers are good at providing feedback to students^2^	220	58	21	21	2.5 (1.0)
30. There are opportunities for me to develop interpersonal skills^4^	222	84	11	5	3.2 (0.8)
31. I have learned a lot about empathy in my profession^3^	219	75	18	7	3.0 (0.9)
32. The teachers provide constructive criticism here^2^	220	56	26	18	2.5 (1.0)
33. I feel comfortable in class socially^4^	222	95	4	1	3.6 (0.6)
34. The atmosphere is relaxed during seminars/tutorials^4^	220	91	6	3	3.4 (0.8)
35. I find the experience disappointing^4^*	217	73	15	12	2.9 (1.0)
36. I am able to concentrate well^4^	221	77	13	10	2.8 (0.9)
37. The teachers give clear examples^2^	222	80	14	6	3.0 (0.8)
38. I am clear about the learning objectives of the course^1^	222	68	21	11	2.8 (1.0)
39. The teachers get angry in class^2^*	222	90	5	5	3.4 (0.8)
40. The teachers are well prepared for their classes^2^	222	94	5	1	3.5 (0.7)
41. My problem-solving skills are being well developed here^3^	222	79	14	7	3.0 (0.9)
42. The enjoyment outweighs the stress of studying physiotherapy^4^	222	81	12	8	3.0 (0.9)
43. The atmosphere motivates me as a learner^4^	222	85	8	7	3.1 (0.9)
44. The teaching encourages me to be an active learner^1^	222	86	10	4	3.2 (0.8)
45. Much of what I have to learn seems relevant to a career in physiotherapy^3^	222	88	6	6	3.3 (0.9)
46. My accommodation is pleasant^5^	222	90	5	5	3.5 (0.8)
47. Long-term learning is emphasized over short-term learning^1^	221	73	19	8	3.0 (1.0)
48. The teaching is too teacher-centered^1^*	222	50	31	19	2.4 (1.0)
49. I feel able to ask the questions I want^4^	222	79	13	8	3.1 (1.0)
50. The students irritate the teachers^2^*	222	78	16	6	3.1 (0.9)

Notes: Negative items, where scores have been reversed, are marked with an asterisk (*).

Item scores that indicate problematic areas (score ≤ 2) and items scoring < 50% Agree/Strongly agree, > 30% Unsure, > 20% Disagree/Strongly disagree are marked in bold.

Superscripted numbers designate the subscale to which the item belongs: ^1^SPL, ^2^SPT, ^3^SASP, ^4^SPA and ^5^ SSSP.

### Demographic distinctions

#### Students’ perceptions of the educational environment – Term

The overall and subscale scores were derived for all the terms on an individual basis and are summarized in Table 2. Terms 1 and 2 scored 78%, terms 3 and 5 scored 74%, and term 4 scored 68% of the maximum score. The terms differed significantly from each other both in relation to the overall score (p = 0.002) and the subscale scores (SPL p = 0.000; SPT p = 0.000; SPA p = 0.011; SSSP p = 0.000). When multiple comparisons were used, they revealed that term 4 students differed statistically significantly; this was most conspicuous compared to students from earlier terms.

#### Students’ perceptions of the educational environment – Gender

The overall mean score was 150.5 (SD 20.8) for females and 148.5 (SD 25.5) for males. There were no statistically significant differences between the groups with regards to the overall or subscale scores. Three items differed significantly between females and males: item 13, “The teaching is student-centred” (3.2 ±0.8 vs. 2.9 ±0.8; p = 0.020); item 15, “I have good friends in this school” (3.6 ±0.7 vs. 3.3 ±1.0; p = 0.008) and item 27, “I am able to memorize all I need” (2.3 ±1.0 vs. 2.7 ±0.9; p = 0.001).

#### Students’ perceptions of the educational environment – Age

Dividing the participants into three groups yielded a mean of 151.5 (SD 21.7) for less than or equal to 21 years, 150.5 (SD 21.2) for 22 to 25 years and 147.7 (SD 23.6) for greater than or equal to 26 years. There were no statistically significant differences between the groups with respect to overall or subscale scores. When the participants were dichotomized as less than or equal to 23 years and greater than or equal to 24 years using an arbitrary cut-off point close to the mean, three items differed significantly between younger and older students: item 9, “The teachers are authoritarian” (1.8 ±1.1 vs. 2.1 ±1.1; p=0.029); item 15, “I have good friends in this school” (3.7±0.7 vs. 3.4±0.9; p=0.004) and item 25, “The teaching overemphasizes factual learning” (1.8 ±1.0 vs. 2.0 ±1.1; p = 0.042).

#### Students’ perceptions of the educational environment – Immigrant background

With regards to participants’ immigrant background, nine students (4%) reported being born outside of a Nordic country, and seven students (3%) reported having both parents being born in a country outside of a Nordic country. Because of the small subsample, no statistical analysis was conducted.

#### Students’ perceptions of the educational environment – Resitting exams

Three groups were identified with regards to the variable “Do you often have to resit exams?” The overall mean score for participants who responded “from time to time” was145.6 (SD 22.3), “infrequently” was 150.3 (SD 18.9) and “never” was 150.4 (SD 24.7). The groups displayed no statistically significant differences in the overall or subscale scores. To facilitate inferential analysis, the groups were divided into two clusters: “I have never had to resit an exam” and “I have had to resit an exam.” This dichotomization did not exhibit significant differences. Five items yielded significant differences between the groups: item 2, “The teachers are knowledgeable” (3.5 ±0.7 vs. 3.7 ±0.6; p = 0.045); item 5, “Learning strategies which worked for me before continue to work for me now” (3.0 ±0.9 vs. 2.7 ±0.8; p = 0.002); item 10, “I am confident about passing this year” (3.2 ±1.1 vs. 3.0 ±1.0; p = 0.003); item 26, “Last year’s work has been a good preparation for this year’s work” (3.0 ±1.0 vs. 2.8 ±0.9; p = 0.029) and item 36, “I am able to concentrate well” (2.9 ±1.0 vs. 2.8 ±0.8; p = 0.017).

#### Students’ perceptions of the educational environment – Previous experience of higher education

The mean overall score was 152.6 (SD 19.6) for those students who had no prior experience of higher education studies and 147.2 (SD 23.9) for those who did. There was a statistically significant lower mean value for the SPA subscale among those who had earlier experience of university studies (38.4 ±5.7 vs. 36.8. ±6.0; p = 0.028). The overall score and other subscales exhibited no significant differences. Three items deviated significantly between participants who had no past experience of higher education and those who did: item 12, “This school is well timetabled” (3.0 ±0.8 vs. 2.6 ±1.0; p = 0.007); item 15, “I have good friends in this school” (3.7 ±0.7 vs. 3.4 ±0.9; p = 0.034) and item 28, “I seldom feel lonely” (3.4 ±0.9 vs. 3.1 ±1.1; p = 0.020).

#### Perceptions of the educational environment – Intent upon completing degree

Dividing the participants into three groups revealed an overall mean score of 150.4 (SD 21.0) among those who intended to work, 150.5 (SD 27.5) among those who intended to continue with higher education studies and 150.9 (SD 20.8) among those who intended to combine work with higher education studies. Participants who responded “working with something else” (n = 7) or “other” (n = 2) were not included in the analysis due to the small size of the subsample. To further assist the analysis, the three groups were dichotomized into two clusters: “work as physiotherapist” and “work as physiotherapist and/or study.” This dichotomization showed no significant differences in the overall or subscale scores. Only one item demonstrated a significant difference: item 39, “Teachers get angry in class” (3.3 ±0.9 vs. 3.6 ±0.7; p = 0.013).

### The open-ended question

Sixty-one (27%) participants responded to the open-ended question. These participants, 49 (80%) women and 12 (20%) men, had a mean age of 26 and were evenly distributed among the 5 terms. A manifest content analysis of the open-ended question yielded the following six themes:

- **Deficiency in the physical environment:** Participants indicated the importance of sufficient and functional lighting, ventilation, working and studying areas and social spaces.
- **Lack of practical training:** Participants, primarily from the later terms, highlighted a shortage of practical training or time allocated to practicing, cultivating and improving psychomotor skills.
- **Pedagogical diversity and percipiency:** Participants desired that teachers use a greater variety of teaching strategies to stimulate interaction during class. Participants, mostly from earlier terms, emphasized that some teachers were not sensitive to the needs of individuals, were perceived as authoritarian and became angry in class.
- **Factual cramming:** An overload of facts and information to be digested over a very short space of time was perceived to bring about stress, frustration and discontent.
- **Autonomy and time for reflection:** Many participants perceived that the study tempo was very high with too many compulsory sessions and an insufficient amount of time assigned to self-study and reflection.
- **Inadequate organization and information:** Participants emphasized that classes were too big; there was a lack of sufficient curricular and extracurricular information and deficiencies in the way in which this was communicated.

### Non-response analysis

Only completed items were included in the analysis, and no imputations were conducted. The non-respondent group included 40 participants (18%) who failed to complete all 50 DREEM items and displayed missing values on at least one of the five subscales. These participants were slightly older (respondents: 24.2 years; non-respondents: 27.0 years), attended term 2 (respondents: 17.5%; non-respondents: 37.5%) and from a country other than Sweden or other Nordic countries (respondents: 2.7%; non-respondents: 10.0%), However, the participants’ responses did not differ from those of the respondents with regards to overall or subscale scores.

## Discussion

Our study set out to explore the educational environment as perceived by undergraduate physiotherapy students. With an overall DREEM score of 150/200 (75%), the students rated the educational environment in this institution as “more positive than negative” and as a marginally “excellent environment”. To better delineate the strengths and weaknesses, the subscales and corresponding items were comparatively interpreted according to the work of McAleer and Roff.^34^ Our study indicated that students perceived teaching positively (73%); that teachers were moving in the right direction (74%); that they were positive about their academic success (72%); that they had a good overall feeling about the atmosphere (78%) and that they had very good social self-perceptions (78%).

Two items consistently received deprived scores, which indicated cause for concern. These statements concerned teachers being authoritarian and teaching that overemphasized factual learning. Students in term 4 differed significantly from those in other terms with regards to overall and subscale scores. Students with prior university education experience perceived the atmosphere more negatively than their counterparts. There were no differences in overall or subscale scores with regards to other variables, such as gender, age, resitting exams or intent upon completing the physiotherapy degree.

An examination of the items on the five subscales and of the inventory as a whole revealed a satisfactory level of internal consistency. The internal consistency of the inventory was above the norm^35^ and similar to published studies.^20^^,^^23^^,^^24^^,^^37^^-^^39^ This implies that the instrument supported the choice of the tool and can be used reliably in the context of physiotherapy education.

Over the past decade, a limited number of DREEM studies have been conducted in contexts that are analogous to the present study (Table 4). While the current study did not set out to compare these studies, they have produced similar results except for the Canadian study by Till,^4^ which reported much lower overall and subscale scores. Of course, contextual dissimilarities, diverse professions and different sample sizes make comparisons difficult. However, while contrasting and interpreting the scores against the guidelines proposed by the developers of the DREEM instrument, certain common trends in the data emerged from the students in these studies, such as being more positive than negative about the general educational environment thereby having an optimistic view of their learning situation, perceiving that teachers are in need of more versatile pedagogical strategies, being academically confident and having a good overall feeling of the educational atmosphere and their social situation.

**Table 4:** Maximum percentage scores of current study compared with similar investigations according to location and/or educational profile

Author	Till	Edgren et al.		Palmgren and Chandratilake	Brown et al.	Ousey et al.	Palmgren et al.
Year	2004	2010		2011	2011	2013	Current study
Educational profile	Chiropractic	Medicine		Chiropractic	Physiotherapy	Physiotherapy	Physiotherapy
Location	Canada	Sweden		Sweden	Australia	United Kingdom	Sweden
Number of participants	407	201*	194*	124	33	22	222
Subscale % max score							
SPL	40	71	71	77	71	60	73
SPT	55	68	70	77	75	75	74
SASP	47	72	69	78	66	72	72
SPA	52	77	79	79	69	71	78
SSSP	52	71	75	79	71	68	78
Overall	49	72	73	78	71	69	75

*A comparative study of two cohorts at two different points in time and therefore two sets of data are presented.

In our study, there was a deterioration in the perceived educational environment in term 4, and it was found to be poorer, on average, as the study duration increased. However, the scores rose again in term 5 when the students were in the clinical phase. Rothoff et al.^40^ stated that it can be assumed that the perception of an accelerative deterioration of the educational environment is not exclusively due to educational delivery but also to individual factors, such as aging, becoming more autonomous and becoming more critical. Young students’ happiness and contentment in relation to higher educational studies or perhaps even enthusiasm for entering adult life is diminishing. Miles and Leinster^31^ postulated that early enthusiasm appears to decrease for many students during the course of their studies, independent of any tangible negative experiences. Results similar to ours have been shown in both longitudinal and cross-sectional studies in which perceptions of the environment decrease with the time spent in education.^4^^,^^24^^,^^38^^,^^41^^,^^42^ However, other scholars have postulated that the environment remains the same over time,^21^^,^^23^^,^^43^ or only increases in remote cases.^37^

In agreement with others,^31^^,^^40^ another interesting result was that students with prior experience of university education perceived the atmosphere more negatively than their counterparts. Even though this discrepancy was not detected on any other subscales, it is possible that by making comparisons with other experiences, the impression of the current environment may be affected, thereby leading to different expectations. These findings could be taken into account in programme development by designing some of its parts to suit the needs of particular groups.

The near non-appearance of differences among predictor variables, such as gender, age and previous experience of higher education, on the overall and subscale levels may, in our view, be another indicator of a satisfactory educational environment in the institution under scrutiny. Admittedly, this ﬁnding does not eliminate differential treatment entirely as minor differences were detected on an item level, but it does at least demonstrate that different demographic groups do not generally perceive the environment differently from their counterparts.

Many previous studies using the DREEM inventory have reported on perceptions of the educational environment among students with an immigrant background,^24^^,^^40^^,^^44^^,^^45^ and researchers have indicated the importance of evaluating the experience and perception of minority groups in healthcare education.^46^^-^^48^ However, it is unfortunate that a comparison of students’ cultural backgrounds was not possible in the present study. The fact that so few students were from ethnic minority groups is something that should be problematized in a society with inherently diverse cultural backgrounds. It is imperative that the institution under investigation address the issue of diversity and the unequal distribution of students.

Edgren et al.^23^ indicated that DREEM results from the overall perception of the educational environment and the subscales may possibly mask the presence of explicit educational problems and that analysis on an item level is necessary. Concurring with Edgren et al.,^23^ we applied this type of analysis to our data to more fully explore areas of strength and weakness.

In the item-level data analysis, students expressed that teachers were knowledgeable and well prepared for their lessons and that they were in a good social environment with good friends; they felt comfortable socially and had a satisfactory living situation. Similar item-level findings in comparable contexts have been reported earlier.^23^^,^^24^

A substantial group of students perceived that there was too much factual knowledge to memorize, and numerous studies^23^^,^^25^^,^^49^ have reported similar concerns. However, these problems are not insurmountable, and addressing them may help to alleviate the anxiety expressed by students. Younger students perceived a greater emphasis on factual learning than their older peers. One could argue that this might have to do with prior experiences. Younger students have less prior knowledge and experiences to scaffold new facts, thereby making factual learning less meaningful to them, which is consistent with the idea that learning has to be meaningful to the learner.^50^ One could also claim that younger students who are in the early stages of their educational process would view “factual learning” as “overemphasized” and that this would subsequently diminish with the introduction of more clinical hours. However, in our sample, this item scored low in almost all the terms, and similar findings have been reported.^25^^,^^51^ Furthermore, this was indirectly supported as students assigned relatively low scores to teaching being too teacher-centred, which may be congruent with the cramming of factual knowledge. Factual learning is possibly driven by the outline of formative and summative assessments. Learning facts is not problematic as such, but learning facts in isolation from a context in which the facts gain purpose and meaning has been recurrently shown to be inferior; the differences between learning as reproducing facts and learning as meaning-making has been studied extensively.^52^^,^^53^ Davis^54^ argued that in order to improve understanding and preserve what has been learned, teaching has to move away from the memorization of facts and passive learning to promote active and more profound approaches to learning that engage students. Implementing educational methods that take authentic clinical cases as their starting point to facilitate meaning-making and applying knowledge as well as reflection on the differences have been found to do away with both teacher centeredness and the emphasis on factual learning.^23^^,^^49^^,^^55^

Currently, education emphasizes self-directed and lifelong learning. The teacher’s role has changed from being merely an information provider to being a facilitator of knowledge acquisition, attitudes and skills required for learning. However, in contrast to this and similar to many other studies,^4^^,^^22^^,^^56^ we found an overall perception that teachers were authoritarian. This suggests that teachers, as elsewhere, are inclined towards traditional styles of teaching and teacher-centred attitudes and practices. We also detected that younger students differed from older ones on an item level regarding the perception of authoritarianism, and students in the early terms also delineated angry and strict teachers in the open-ended question. Lemp and Seal^57^ reported that students often perceive hierarchical and competitive atmospheres in which haphazard tuition and teaching by humiliation continue to occur. Recognition and reform of the environment are required to achieve the necessary fundamental changes to the culture of undergraduate healthcare professional training. On the other hand, in a subsequent focus group interview with students (not reported here), it emerged that students had difficulty understanding and explaining the connotation of the word “authoritarian”, thereby making this item a possible instrumental artefact and creating measurement noise in the instrument. Furthermore, there may be cultural differences in the meaning of the word. However, this result could also be an accurate interpretation of the students’ perceptions of the environment. It is therefore imperative to remind teachers that respect for students is vital to the learning process.^15^^,^^55^^,^^58^In agreement with other scholars,^22^^,^^30^ both the closed- and open-ended responses revealed that the respondents perceived student support mechanisms to be inadequate. Professional healthcare education students are exposed to a diversity of pressures, many of which may cause stress. These stresses - examinations, rivalry, information overload, time management, financial issues and relationship problems are akin to the pressure encountered by all students.^59^ Creating an adequate, functional and accessible support system may help improve the environment and reduce the attrition rate.Previous scholarly work has shown that students with good exam results assess the environment more positively than those with poorer exam results.^20^^,^^37^ In the present study, we did not correlate the DREEM scores with exam results, but students who disclosed having to resit exams were more positive towards the quality of teachers despite perceiving a low level of confidence and difficulty concentrating. It is possible that underachieving students have not become autonomous, self-directed learners and more extensively adopt a surface approach to learning.^60^ This kind of learning style is predominantly concerned with recollecting facts, memorizing what they have learned and performing well in their assessments. When an educational programme explicitly or implicitly promotes surface learning, it is possible that anxiety, stress and aggravation during learning can progressively increase and lead to deprived performances.^37^As mentioned earlier, we found a discrepancy in the environmental perceptions of students with prior experience of higher education. On an item level, this group of students was also more negative about scheduling, reported that they did not have many good friends and felt more lonely than their peers. Studies have shown that students with prior higher education experience generally have a more negative perception of the newer environments than their counterparts.^31^^,^^40^ These students tend to be older and have more life experience. They may also have other obligations, such as work and family, which make it difﬁcult to socialize outside of structured school hours. It could be viewed as a paradox that those students who have a wider spectrum of educational experiences, and possibly more social networks and obligations, are less positive about the environment as they might not be as dependent on the environment as their peers. Such differences may inﬂuence responses to items, such as gauging friends in the school and having a good social life. Similar tendencies have also been reported by Miles and Leister,^31^ who stated that students with graduate backgrounds perceived the environment more negatively than school-leavers. Unfortunately, in this study, it was not possible to determine whether this result was due to educational background as age might have produced a confounding effect.The open-ended question asked at the end of the DREEM inventory has scarcely been reported in the literature. However, here, this question not only confirmed some key areas identified in the inventory but also captured new areas, for example, deficiencies in the physical environment, the desire for more practical and clinical training, fewer compulsory sessions and lectures and more time for independent study and reflection. These concerns reflect a general perception among students that would probably not have been highlighted had we decided against the use of the open-ended question. Similar findings from qualitative data have been highlighted by others.^61^^,^^62^ It would be fascinating to explore these aspects of the educational environment. To get a more profound understanding of what constitutes an educational environment, we believe that full-scale qualitative explorations are warranted for other aspects of the educational environment that are not captured by the DREEM inventory in focus - for example, emotional aspects, features of mutual dependencies between students and teachers, issues of inequality and organisational and hierarchical aspects.

### Limitations

All students from terms 1 through 5 were invited to participate in the current study. Students in term 6 (the last term) were not included because they were on clinical placements and/or writing their dissertations at the time of the data collection. Thus, one could argue that this non-probability sample did not capture the full spectrum of students in the present context.Two items (6 and 18) in the inventory were concerned with patients, and more than 30% of the participants responded “unsure”. During the analysis, it was evident that students from terms 1 and 2 constituted the majority of these respondents and have had no or very little clinical training, which probably explains this variation. The methods for analysing and reporting data derived from DREEM have not been consistent in the educational research literature, and the manner in which Likert response data should be analysed has been debated.^63^^-^^65^ In their recent methodological study, Swift et al.^66^ provided evidence that when comparing independent samples of Likert response data, non-parametric Wilcoxon-Mann-Whitney tests perform well and may have greater power than parametric t-tests. Furthermore, because skewed distributions often occur in DREEM data, an item with an adequate central measure may mask a high proportion of negative responses. Therefore, we followed newly published guidelines^66^ and merged the agree/strongly agree with the disagree/strongly disagree responses. Swift et al.^66^ also indicated that values below the expected mean, as recommended by the developers, could be a priori raised to 2.5 if researchers wanted to be more stringent in the analysis. However, we chose to follow the guidelines developed by the originators and used 2.0 as the limit.

With regards to the subscales, one could argue that Pearson’s correlation coefﬁcient is best suited when the level of measurement is the interval type and that Spearman’s rank correlation coefficient should be employed when the level of measurement is the ordinal type. However, it is difficult to identify the subscales in the DREEM inventory as more ordinal than interval. Therefore, we also employed Spearman’s correlation (not reported); the results obtained using Spearman’s correlation did not differ from those obtained using Pearson’s correlation.

Despite the large number of studies in which DREEM has been employed, very few psychometric reports have been published since the development of the inventory. More recently, there have been some concerns regarding the psychometric robustness of the instrument.^18^^,^^67^^,^^68^ Replications of the five-factorial structure have only been moderately successful, which indicates some instability in the instrument. The Swedish version of the DREEM instrument has been reported as valid and reliable, except for the factor structure.^18^ Jakobsson et al.^18^ proposed a new five-factor solution for the Swedish version but stated that it had not been proven to be a superior measurement model compared to the original. This calls for future research to continue to explore and determine the psychometric properties of the instrument.

### Implications for future healthcare education research

Recently, Schonrock et al.^69^ reported that many instruments used to assess the educational environment in healthcare education are not grounded in theory. They indicated that the deﬁciency of a theoretical framework may explain the differences regarding the concepts measured in many studies. The reason for this deficiency of theory is probably due to the fact that when scholarly work took off in the late 1950s, researchers were more occupied with attempting to measure the concept rather than trying to conceptualize and theorize it. Indeed, although frequently used in different forms, the notion of educational environment is rarely defined.Student cohorts can fluctuate and be very diverse and distinctive from year to year, which is why we considered it important to include students from several terms in this study. However, further research should consider designing longitudinal studies and collecting data from students over longer periods. In the present study, we investigated students, but the perceptions of faculty and other stakeholders are equally important. Genn^2^^,^^70^ indicated that the organizational environment of teachers is inextricably bound to the educational environment of students and is a strong determinant of the environment. However, as teachers often remain in a teaching position for a long period, it is conceivable that they perceive the educational environment as more consistent. Although this is speculative, it is surprising that teachers’ perceptions of educational environments have only been sparsely investigated.^40^ Thus, there is a need for further research in this area on comparisons between students’ and teachers’ perceptions.The specific aim of the current study did not entail a statistical comparison with previous findings, for example, of chiropractic students. However, our results indicate that there may indeed be several commonalities between the physiotherapy and chiropractic student groups, which should be explored in further studies.The use of a questionnaire to assess the perception of educational environments can be complex because there is the risk of excluding certain explicit elements. DREEM creates a snapshot of perceptions of an educational environment but cannot provide data regarding the concerns underlying poor scores or other constructs that the instrument does not encompass. Using qualitative methods such as focus groups, observations or semi-structured interviews with key stakeholders and/or outliers could be useful to further explore and more fully understand the concept of educational environment.Overall, the students in the present study were positive about their educational environment. Notwithstanding, more research must be conducted to battle the overemphasis on factual knowledge, and there should be further pedagogical and organizational faculty development that extends beyond lecturing and teaching.Thus, the educational environment constitutes internal and external features on microscopic and macroscopic levels as well as formal and informal settings. Nevertheless, a clear definition of the educational environment and its components remains elusive, and there is much to be explored in terms of the factors and concepts involved. However, we can say that research on educational environments probably requires both qualitative and quantitative methodologies, psychometrically sound instruments and an exploration of all stakeholder groups involved in constructing, affecting and influencing the phenomena.

## Conclusion

The results of this study provide valuable clues regarding how undergraduate physiotherapy students perceive their educational environment. Students were positive about the teaching and teachers, were positive about their academic success and had a good overall feeling of the educational atmosphere and their social self-perceptions. Overall, students perceived that the institution provided a sound educational environment and educational programme despite some demographic variations. However, certain explicit elements of the educational process may need to be addressed to further enhance the educational experience.

## 

### Conflict of Interest

The authors declare that they have no conflict of interest.
